# Nursing Section

**Published:** 1904-03-12

**Authors:** 


### The Hospital.

### Nursing Section.

Contributions for this Section of "The Hospital" should be addressed to the Editor, "The Hospital" Nursing Section, 28 & 29 Southampton Street, Strand, London, W.C

No. 911.—Vol. XXXV. *SATURDAY, MARCH* 12, 1904.

### Notes on News from the Nursing World.

### QUEEN ALEXANDRA'S MILITARY NURSING SERVICE.

It is officially intimated that the following staff nurses belonging to Queen Alexandra's Imperial Military Nursing Service have been promoted to the rank of Sisters:—Miss M. M. Blakely, Miss J. A. Evans, Miss A. Fitzgerald, Miss E. C. Humphreys, Miss A. C. Jacob, Miss M. Pedler, Miss M. L. Potter, Miss L. A. Rideout, and Miss M. M. Tunley.

### THE PRINCESS OF WALES AT THE EVELINA HOSPITAL.

On Monday afternoon, the Princess of Wales, attended by Lady Katharine Coke, paid an unofficial visit to the Evelina Hospital for Sick Children. Owing to the private character of the visit, the house officials only were present. Her Royal Highness was received by the matron, Miss Getz, the secretary, Mr. Alfred Yorke, and Dr. Bent, the house surgeon, and after inspecting the new nurses' quarters, proceeded to the wards, and showed much interest in all the little patients, visiting every cot and talking to all those who were able to understand. The Princess greatly admired the blue and white tiling of the wards, the red twill bedgowns and the red ward tables, commenting on the general cheerful aspect of the hospital. One little girl of three who had had her foot amputated only a week previously, "gave a song," and all the children, although fully alive to the fact who the visitor was, were as happy and natural as possible, especially a little boy of four who "hung on" to the Princess in a most unceremonious manner. Her Royal Highness spoke a few words of comfort to one of the patients' mothers, whose child was in a very critical condition. After spending an hour in the wards, and giving joy to all, the Princess rested for a short time in the matron's room, and before leaving, signed her name in the visitors' book. On stepping into her carriage her Royal Highness received a hearty cheer from the ragged youngsters of the neighbourhood, who had collected in full force. The Princess further showed her sympathy and kind interest, in sending a telephone message to the hospital on Tuesday morning, asking to be informed of the condition of the child whom she found in such a critical condition the day before. Fortunately the doctor was able to report a slight improvement.

### PRINCESS CHRISTIAN AND THE NURSES OF THE ROYAL FREE HOSPITAL.

By the desire of Princess Christian, the President of the Royal Free Hospital, the proceeds of the bazaar held last June at the Prince's Skating Rink are to be devoted to the improvement of the nursing quarters. The bazaar, which was organised by Princess Christian and Princess Louise Augusta, yielded the sum of £3,166 18s. 2d. The intended alterations will not have the effect of adding to the present sleeping accommodation, but the existing rooms will be greatly improved. Six of the double-bedded will be converted into single rooms, while the whole of the 30 rooms on the top floor will be made accessible by means of an electric lift. A sisters' common-room is to be built on the roof of the south wing, where breakfast, tea, and supper will be served. The remainder of the available space will be devoted to a promenade for the nurses. The Princess has throughout taken the greatest interest in the plans, and the fittings, furniture, and decorations will be chosen under her personal direction.

### LADY DUDLEY'S NURSES' FUND.

A list of subscriptions to the Countess of Dudley's fund for the establishment of district nurses in the poorest parts of Ireland, just published, shows the general interest which her efforts are exciting. In particular, the assistance tendered by the Countess of Mayo merits recognition. Lady Mayo has been collecting money in various counties, with the result that she obtained in subscriptions and donations £55 in Waterford, £14 5s. in King's County, £37 5s. in Kerry, £19 17s. in Sligo, £47 7s. in Roscommon, £10 2s. in Leitrim, and £6 11s. 6d. in Longford. Some of this money was contributed by personal friends, but a considerable proportion was given in small sums. With a few such helpers as Lady Mayo, the wife of the Lord Lieutenant should soon not be far from fulfilling her ideal, which is to provide a nurse for every district in which she is needed.

### PRIVATE NURSING IN RUSSIA.

The account in another page of the experiences of an English nurse in Russia will be perused with special interest at the present time. With regard to an operation which took place on one of her little patients in the doctor's home, she states that there were no startling divergencies from methods obtaining in this country, and it appears from the details she gives that the home was admirably managed. There is one little lesson to be learnt from her story. Suffering from the bites of mosquitoes, she tried on herself the same remedy which had proved efficacious in the case of her patient, with the result that blood-poisoning set in. Happily, it was localised by the care and skill of the doctor, who, however, very properly administered a gentle reprimand to the nurse for not consulting him instead of herself trying to relieve the irritation.

### A HANDBOOK FOR MIDWIVES.

"A COMPLETE Handbook of Midwives," by Dr. J. K. Watson, M.D., will shortly be published by The Scientific Press, Limited. This handbook has been designed to meet the requirements of the Central Midwives Board, and is an up-to date text-book of all that the midwife requires to know of the subject treated. The work is produced with the object of rendering it unnecessary for midwives, during their preparation for examination, to consult the larger and more expensive works on the subject written especially for medical men. The illustrations are very complete, numbering some 130 separate diagrams, most of which have been specially prepared for the book.

### THE SUPERINTENDENT NURSE OF LADYWELL WORKHOUSE.

AT the meeting of the St. Olave's Board of Guardians on Thursday last week the duties of the superintendent nurse were arranged in accordance with the wishes of the medical officer of Ladywell Workhouse. We hope that this concession on the part of the Guardians, which we urged a few weeks ago, will be followed up, at an early date, by the addition of trained nurses, who are at present represented by the superintendent nurse alone, to the staff.

### BLANKETS AND PILLOWS AT NETLEY.

AN inaccurate statement is made in the columns of a weekly contemporary respecting a recent incident at Netley Hospital. It is complained that while an orderly who lately lost some blankets has had to pay for them, a sister who lost some feather pillows refused to pay for them, and they were therefore charged upon the detachment. The fact is that the regulations at Netley provide that when an orderly loses a blanket he pays for it, but that if feather pillows are lost from the wards generally the whole detachment must pay for them. There cannot in the latter case be any question of a sister "refusing to pay," as she is not expected to do so.

### A REPROACH TO THE SOUTHAMPTON CHURCHES.

BOTH in the report and at the annual meeting of the Southampton Branch of Queen Victoria Jubilee Nurses' Institute the failure of the clergy and ministers of religion to give substantial support to the movement was alluded to. The Chairman, in his speech, mentioned that the parishes of St. Michael's, All Saints', St. Matthew's, and Holy Trinity contributed nothing at all, whilst the amount received from the other two churches was less than £2. This is really a reproach to the Southampton churches, and we hope that they will make haste to remove it. There is no question as to the excellence of the work done by the Queen's Nurses in the town. Last year they paid upwards of 13,000 visits, or 1,000 more than in 1902; and though there have necessarily been changes in the personnel, it is universally admitted that complete satisfaction has been given by the lady superintendent and her staff. The only weak point of the report is the fact that there is a balance of £24 on the wrong side, subscriptions and offertories, the latter especially, having considerably fallen off. We entirely agree with the Chairman that it is not much to the credit of Southampton that it can only raise £124 for the maintenance of the Queen's Nurses.

### FREE OR ASSISTED MIDWIFERY TRAINING.

UNDER the auspices of the Association for Promoting the Training and Supply of Midwives, 12 Buckingham Street, Strand, a home has been opened at East Ham, in co-operation with the Plaistow Maternity Charity, where four pupils are trained in a crowded and very poor district, under a competent midwife, for the certificate of the Central Midwives Board. The demands for the services of the midwife are, it is stated, at times almost more numerous than she can cope with, and the Association feels that, although so limited in extent, a most useful work is being done in ministering to the needs of a very poor district, as well as in the opportunities of training which are thus afforded. But the committee desire to largely extend their operations; and they therefore invite applications from superior women willing to take up district midwifery work in order to arrange for their training in approved hospitals and other institutions and centres. Money is, of course, needed to enable them to carry out their plans, and they appeal for annual subscriptions to all who recognise the importance of the movement.

### DEATH OF A WELL-KNOWN IRISH NURSE.

THE nursing world in Ireland has sustained a severe loss by the death of Miss Eva Maguire, who contracted typhoid fever when she was nursing the patients in the wards of the Hardwicke Fever Hospital at Dublin. Miss Maguire had been for nine years attached to the nursing staff of the Home of Industry Hospitals, and for some time had been sister in-charge of the fever department. The utmost confidence was felt in her judgment by the medical staff, and she was absolutely devoted to her work. In fact, her tender and sympathetic personality endeared her to everyone. Her remains were laid to rest in Glasnevin Cemetery, and a remarkably representative gathering of doctors and nurses attended the funeral, including the lady superintendent and several sisters of the hospitals in which she spent the best years of her life. Many wreaths were sent by attached friends.

### TROUBLE AT AN ISOLATION HOSPITAL.

THE matron of the Isolation Hospital at East Ham, who has held her present position for ten years, has received a letter from the Clerk to the East Ham District Council requesting her to resign her position. We understand that certain allegations have been made reflecting upon her management, but that she has not been allowed to appear before the Council to answer them. On the other hand, it seems that three members went to see her at the hospital, but she was not well enough to enter into an explanation.

### DISTRICT NURSES AND PAYING PATIENTS.

THERE is a difficulty at Port Talbot about obtaining a nurse for paying patients. It has been suggested that the Queen's nurses should receive fees from persons who can afford to pay. This suggestion was made at the annual meeting of the Aberavon and Port Talbot District Nursing Association by Dr. Williams, who complained that the time of the two nurses was very much taken up in attending fairly well-to-do people able to pay for a private nurse, but unwilling to do so as long as they were attended gratuitously. It is curious that such a proposal should be brought forward in face of the fact that the Port Talbot Association is affiliated to Queen Victoria's Jubilee Institute, whose nurses are not allowed to make a charge. On the other hand, it is also against the rules of the Institute that they should attend any but the sick poor. We think that if, as we understand, there are only a small number of patients who could afford to pay a fee and not sufficient to maintain a visiting nurse in work, the difficulty might possibly be met by a member of the committee of the association looking over the list of patients every week and writing at once to those not strictly belonging to the sick poor asking them to contribute a special donation. Of course, there ought to be no need to prefer the request, but as there apparently is, it should not be postponed until the patient is well and consequently has forgotten the debt of gratitude due to the nurse.

### A NEW TRAINING SCHOOL.

The West Bromwich Guardians have received a letter from the Local Government Board stating that they are prepared to recognise the West Bromwich Workhouse Infirmary as a training school for nurses within the meaning of the Nursing Order of 1897. At the suggestion of the Board, the Guardians have decided that the certificates granted to nurses as to efficiency and conduct at the expiration of three years shall be given upon the combined reports of the medical officers and the superintendent nurse, and also that the examinations shall be conducted by an independent examiner.

### THE MIDWIFE FOR MIDWIFERY CASES.

The medical officer of Eastville Workhouse has reported to the Bristol guardians that two women in the lying-in wards were suffering from septic infection. He affirms that there will always be a risk of lying-in women becoming infected in this way when nurses who are allowed to attend midwifery patients are dressing septic cases. Therefore he holds that no nurse should attend midwifery cases unless she can give her whole time to the work. The question has been referred to the Hospital Committee, and its importance will be recognised. If it be not always practicable for a nurse to give her whole time to midwifery work, it should be quite possible for one who leaves an ordinary ward in order to assist in the lying-in ward to take such precautions that she is not likely to convey septic infection.

### DISTRICT NURSING IN DEVONSHIRE.

Good work continues to be done by the District Nursing Associations in Devonshire. At Exmouth, where the Association last year had a balance of 2d. on the right side, the total receipts were £105 3s. 5d. and the district nurse made 5,472 visits to 214 patients. At Crediton there is an adverse balance of £45 12s. 2d. due to the maternity branch, but £50 is to be drawn from the reserve fund to square it. At Bideford the nurse attended 117 cases and paid 2,472 visits, but the credit balance has been reduced from £14 6s. 9d. to £1 5s. 6d. At Ashburton the average number of weekly visits was 73, and there were 95 patients. The late nurse was only paid £52, but the present one receives £76, so that the Committee have to make an extra endeavour to raise subscriptions. At Chudleigh the nurse, who formerly held the appointment and resigned owing to ill health, has again taken up the work and been cordially welcomed after five months' absence. In this instance, 1,753 visits were made and the total expenses for the year were £49 5s. 8d.

### VILLAGE NURSING.

The secretary of a branch of the Holt-Ockley system protests against the expression "untrained nurses" as applied to the Holt-Ockley nurses which occurs in an article on "Village Nursing" in a well-known periodical, and declares that the training they receive for four or six months in the poor districts of London fits them for cottage nursing better than a longer period in a hospital. She is justified in her protest. The proper description is partially-trained nurses, but though partially-trained nurses are preferable to none at all, it stands to reason that, other things being equal, fully-trained nurses are the best, whether for nursing in mansions or in cottages.

### A SERIOUS DEFICIT AT WALSALL.

At the annual meeting of the Walsall Victoria Nursing Institution the very disappointing announcement was made that the financial support given to the Association during the past year had been quite inadequate, and that the balance on the wrong side was just over £100. The Chairman, in moving the adoption of the report, said that in order to continue the work satisfactorily at least £200 a year more was required, and it was suggested that towards this the Walsall guardians should be asked to contribute £25 a year in addition to the contribution of £25 already made. As 9,915 visits were paid by the nurses to the sick poor, against 7,994 in 1902, the proposal merits favourable consideration. We observe that the private branch of the Institution, instead of being, as some expected, a source of income to the district branch, is not profitable. If it is not always clearly brought out by collectors of subscriptions that all money subscribed by the public is expended upon district work only, the difficulty experienced in obtaining the money needed by the Institution may be partly accounted for.

### STOKE-UPON-TRENT UNION HOSPITAL.

The medical officer of health for Stoke-upon-Trent, Dr. Petgrave Johnson, recently conducted an examination for nurses of the Stoke Union Hospital seeking qualifying certificates. Nurses L. M. Young, S. S. Swift, L. Towl, L. Till, and K. Marson were successful. Miss Young and Miss Swift also possess the certificate of the London Obstetrical Society.

### SHORT ITEMS.

A meeting will be held at the Mansion House on Monday afternoon at 3 o'clock, on behalf of the proposed National Training School for District Midwives, Lady Stanfield in the chair. The speakers will include the Bishop of London, Miss Annie McCall, M.D., and Miss Alice Gregory.—The amount realised at the recent ball in aid of the Queen Victoria Nursing Institution at Wolverhampton was £45.

### The Nursing Outlook.

"From magnanimity, all fear above;
From nobler recompense, above applause,
Which owes to man's short outlook all its charm.'

### NURSING IN GREAT BRITAIN.

### I.—Fifty Years' Development.

England is justly proud of Miss Nightingale, for Miss Nightingale is the mother of reforms which, in decades of years, have produced the modern nurse. Although well stricken in years, and a confirmed invalid, Miss Nightingale has never failed to exercise influence in connection with the system of training and policy pursued by the first training school in England, which, in 1857, was founded in her honour by public subscription. A great many things have happened in the period of nearly fifty years which has intervened since Miss Nightingale applied the money given her as a testimonial to establish a training school for nurses. The work for the first fifteen years was greatly handicapped by the difficulty of obtaining suitable women to train as nurses. In those days a hospital was merely regarded as a place to which poor sufferers and accidents could be sent for treatment. The hospital was not popular with the people of any class, the mortality was often appallingly high, and the risks to the patient from hospitalism were serious. There was really no nursing worthy of the name in the modern sense. The nurses were, for the most part, uneducated and untrained, the moral tone was low or often wanting altogether, the accommodation for the nursing staff in many cases was shameful and nearly always insanitary, whilst the food was inadequate, of poor quality, and badly served.

When the writer first took responsible charge of a large hospital in 1868, most of the nurses slept in the basement, in damp and ill-ventilated rooms, some with brick or stone floors, and in all the light was very defective. These rooms were in fact cellar dwellings. The day nurses were nearly all old women of the charwoman class, and there was no regular staff of night nurses at all. A very large proportion of them could not write their names, and had to make a cross when they received their wages. The night nursing was usually done in those days by old women, who consented to sleep in the hospital chairs instead of in their beds at home, for which service they received 1s. 6d. a night, or about 10s. a week. Nearly everywhere this singular state of things prevailed, and in one of the largest London hospitals the nurses were placed in cupboards or dens on the staircases, where neither quiet, privacy, proper air, nor light could be obtained. So recently as 1871 a leading hospital authority defended the system which made each nurse scrub the floors of her ward, on the ground that if the scrubbing were to be discontinued there would be really little or nothing for the nurses to do. This fact will more forcibly illustrate the condition of matters in regard to the nursing some 30 years ago than anything which can be written.

No wonder, then, that Mrs. Wardroper, of St. Thomas's Hospital, and other leaders in nursing matters in those days, found it difficult or impossible to secure applications from a sufficient number of suitable women to be trained as nurses. It took another 10 years of hard work to improve the accommodation, amend the system, change the principles of treatment whereby hospital mortality was reduced after major operations from 33 per cent. to less than 3 per cent., and so to gradually attract young women of a better class to offer themselves for training as nurses in hospitals. This stage was reached some 20 years ago, and since then a revolution has taken place in hospital nursing in this country, and indeed throughout the world. To-day, in the well-administered hospitals, nurses have everything of the best; they are trained in all the duties of their calling during a period of at least three years; their knowledge is tested by periodical examinations, and at the end of the period they receive a certificate of efficiency which qualifies them to earn at least £100 a year as a private nurse, or the equivalent of some three-fourths of that amount by working in a hospital or institution. They are housed in special buildings replete with everything calculated to provide for health, exercise, and comfort; their food, hours of work and relaxation are carefully regulated, and to a woman of character the nursing profession of to-day offers a career full of promise and happiness.

Not only does nursing offer a career for women at the present day, but the nurse's calling has been elevated into a profession, and claims recognition by the legislature. Twenty years ago, however, Miss Sherriff and other leaders of women ridiculed the idea that nursing could ever offer a career for gentlewomen. It is true that as the numbers of trained nurses have increased, the difficulty of selecting suitable women for the higher offices has increased also. We believe, however, that with the aid of Bedford College and the rest of the training schools this difficulty will gradually diminish.

It will thus be seen that Miss Nightingale is the woman of the nineteenth century, for she has effected a social revolution in every civilised country throughout the world without bloodshed or commotion. Indeed, the quietness of her methods is apt to make us overlook the radical character of the reforms resulting from her example, life and work. We propose to show the effect of this revolution upon the nurses' training schools, the organisation of nurses and the societies connected with them, and also upon the status and position of the individual nurse *vis à vis* with the public.

### Lectures Upon the Nursing of Mental Diseases.

By Robert Jones, M.D.Lond., B.S., F.R.C.S.Eng., M.R.C.P.Lond., Resident Physician and Superintendent of the London County Asylum, Claybury.

### LECTURE VII.

Reference was made in Lecture IV. to the balance between repair and waste, to the continuous assimilation and destruction which proceed—so to speak—simultaneously in the human organism, the processes being attended by the liberation of energy and the production of heat. In Lecture V. and VI. we dealt with "nutrition" the building up of protoplasm, through the agency of the physiological processes of absorption, digestion, and respiration. These functions constitute one-half of metabolism, the other half being concerned with the excretion of useless and waste material not required in the process of growth. In addition to the lungs, as organs of excretion for the discharge of carbon dioxide (carbonic acid gas) and aqueous vapour, there are also the kidneys and the skin. All three organs eliminate a certain amount of water with various salts in solution. While the lungs expire, the skin exudes and the kidneys excrete noxious waste material. The kidneys, consisting of long, coiled tubes which are lined with cells, perform a double function. They act in part as filters, in part also as secretory organs for urea—the great waste product of the tissues—and mineral salts which are yielded direct from the blood. Thus the kidneys perform a physical as well as a physiological office. Moreover, it has been proved that they control the waste of the tissues, besides excreting the urea. The blood from the renal arteries, which are very large, flows into the capillaries, these terminate in the outer part, or cortex of the kidney in exceedingly small clusters called *glomeruli*, so that each glomerulus has a little artery, a loop of capillaries and a little vein, but after the vein emerges it breaks up again into a further set of capillaries round the convoluted tubes. These tangled knobs of blood-vessels in the glomeruli are pushed up against the end of a tubule which then stretches over it like a little cap, hence called a *capsule*, and as the pressure of the blood is greater in the capillaries than it is within the capsule of the kidney tube, an oozing or filtration takes place, and the watery constituents of the blood, with certain soluble salts, pass through the renal tubules into the pelvis of the kidney, and thence along the ureter to the bladder—where they are accumulated as urine until voided.

The tubules of the kidney are exceedingly fine thread-like pipes, and their course through the kidney itself is peculiar. Beginning round the glomeruli in the cortex or outer part—seen to be dark brown by the naked eye—where they coil and twist, they run down the medulla or striated inner portion of the kidney, then turn back to the cortex—where they are surrounded by the capillaries from the dividing little veins of the glomerulus already mentioned—here again they loop and come straight down the medulla into the pelvis of the kidney. These tubes in their course have two kinds of epithelial cells lining their walls. Those round the glomeruli (formed, as stated, of a cluster of capillaries) are flat, and they act as filters. The other cells lining the tubes lower down are spherical or cubical in shape. It is these that especially secrete the urea and other substances from the blood. These cells of the kidneys do not manufacture urea from the blood. This has already been done by the liver cells from the ammonia salts which are given out into the blood by the muscles and other protoplasmic tissues. Urea exists in the blood (although in very small quantities) before it circulates in the kidneys, and is collected by the cells of the kidneys, which pick it out, so to speak, as the blood circulates in them, for urea can accumulate in the blood after both kidneys are removed. It is not filtered or diffused through these cells. Urine contains a hundred times more urea than the same volume of normal blood. In disease of the kidneys these cubical cells do not act efficiently, and blood, albumen, bile, or sugar may in consequence pass into the urine.

The quantity of urine secreted varies much. In winter the vaso-motor nerves cause the vessels of the skin to contract, preventing loss of heat, and the blood from the surface of the body flows in consequence into the interior of the body, and the kidneys, owing to this greater blood flow, secrete more freely. In summer, on the contrary, the kidneys are less active, but the skin more so. Drinking fluids causes more secretion from the kidneys, for it increases the flow of blood through them. Certain medicines when absorbed into the blood excite the cells of the tubules to secrete; these are called *diuretics*. Other medicines may act on the heart or the blood-vessels, raising the pressure within them and so promoting an increased flow of urine.

When the blood stops for a long time in the veins and the circulation is retarded—as occurs in disease of the heart—the water and soluble proteids of the blood ooze out or filter from the capillaries into the tissues, causing dropsy. When this occurs measures are taken to get the skin to act more freely and to compensate for the deficiency in the kidneys.

When the urine reaches the bladder it accumulates there until this receptacle is full. The distended bladder in sane persons excites the nervous system to put into action a certain mechanism by means of which the urine is voided. The sensory nerves in the wall of the bladder are stimulated into action by the distension; this stimulus is carried up by the afferent nerves to a spinal reflex centre in the spinal cord from which an efferent impulse is dispatched along other nerve fibres causing the sphincter muscle of the bladder to relax and the muscular wall to contract, when the urine is expelled and the bladder emptied. This sensation extends to the brain in healthy persons and is under the control of the will. When certain great tracts of nerve fibres are destroyed through disease of the spinal cord, or when the mind is affected through disease of the brain, the person loses control over the sphincters of the bowel and the bladder, and these organs void their contents upon unfitting occasions and without rousing consciousness. Patients thus afflicted should never be reproved for what they cannot prevent. It is a symptom of deficient nervous control, and care should be taken that they are properly attended to so often as the occasion for this occurs. Care should be taken that the voiding of urine—the "dribbling" as it is called—is not the effect of over distension of the bladder. The bladder is sensitive to the smallest accumulation of urine in healthy persons, but in cases of general paralysis of the insane and the dementia resulting from epilepsy, as also in cases of profound melancholia, the senses become so blunted that the patient is unaware of the accumulation, or if aware may not have the intelligence to set the nervous mechanism into action to expel the contents. Serious and fatal consequences may take place, and have, taken place in patients thus affected, through rupture of the bladder. In many mental cases the nurse has to be most alert and attentive to change patients wetting their bed or body linen, more especially in helpless, bedridden, feeble, terminal cases of insanity. Melancholy and demented patients should be raised at fixed intervals during the night. It is well to encourage mental patients, who are not too feeble, to void urine at stated intervals—upon rising in the morning after each meal, and at bed time, as it is by repeated attention to habits of body that self-neglectful patients may be roused to decency and self-respect. Such attention not infrequently starts regular processes of thought which serve as motives for conduct, and such attention becomes an important part of a nurse's duty when nursing mental cases. Care to keep such patients clean prolongs their lives, and when bedridden it prevents the formation of bedsores, and, in consequence, death from blood-poisoning through the invasion of bacteria acting upon the sodden and broken skin. The nurse should never attempt to draw off the urine from a male patient, but she is expected to use the catheter in those of her own sex.

The skin, another important secretory organ is developed morphologically from the same layer of embryonic tissue whence also the brain grows. Every portion of the skin is represented centrally in the brain. The skin has an upper or superficial layer of *epidermis* or cuticle, and an inner one, the *dermis* or true skin. The outer, which is bloodless, is subdivided into the scaly, hard, horny or *corneous* dead cell layer, and the inner, the *Malpighian* which, owing to the deposit in it of pigment or colour—gives the blackness to the skin of the negro. The dermis is ridged into folds, contains blood-vessels (capillaries) which make it extremely vascular. It is from these capillaries that lymph exudes to nourish the soft under-surface of the epidermis, and it is owing to the exudation from damaged capillaries that blisters occur after burns, injuries, and through nervous influences in the last stages of general paralysis. The dermis is the seat of the nerve endings or *touch-corpuscles*, which give the power to estimate sensations of touch, such as, rough or smooth, hard or soft, sharp or blunt, and heat and cold. Beneath the dermis is a layer of fat, which acts in part as a soft cushion, and in part as a non-conductor to prevent the loss of heat. Underneath the fat are the muscles encased in connective tissue-sheaths. The dermis is thick on the back and shoulders to adapt it for carrying burdens, but on the tips of the fingers it is very thin. The skin bears hairs in the hair follicles, and nails on the digits; and all of these may reflect special conditions of the nervous system. The hair may turn white suddenly, or gradually, or may be shed through emotions of fear, grief, or other nervous influences. It is asserted that partial and even complete baldness occurs less through nervous influences than from the invasion of the hair follicles by hurtful parasitic bacteria. Each hair has a small secreting gland (the *sebaceous*) which pours out into the follicle an artificial pomade to keep the hair-shaft smooth and glossy. Each hair has (markedly in some animals) a small muscle fibre (the *pilo-motor*) attached to the follicle, which enables such animals to raise the hair erect under the influence of emotion. This feature may occasionally be noticed in man. The other gland of the skin are the sweat glands (*sudoriporous*). They open by the so-called "pores" of the skin in the intervals between the little ridges of the epidermis; and in the palm of the hand there are no less than 3,000 of these in every square inch of the skin. It is calculated that in every person there are about two millions of these glands, and that their tubes would extend to a distance of 10 miles if placed end to end. The ridges referred to have a peculiar appearance on the pulp of the finger tips, being arranged in concentric whorls which never change their form, and which has enabled them to be used as a safe and reliable method for the identification of criminals. The secretion of the sudoriporous glands is the perspiration. It is chiefly water and common salt, with traces only of some other bodies. There is a deterioration in the secretions of the skin in insanity, which is made evident to the sense of smell, although the patient be well bathed and cleansed with soap and water. Carbon dioxide likewise occurs in the perspiration as it also occurs in the urine, saliva, and bile. It is estimated that one-fiftieth part of the total carbon dioxide excreted is discharged by the skin. The skin can allow of the passage of water (and also of some salts) from without, inwards into the blood, but the quantity is exceedingly small unless the epidermis is removed, for sailors immersed in salt water may die of thirst, and we are able to handle poisons freely if the skin is unbroken. The perspiration is secreted continuously and passes unconsciously by evaporation from the "pores" into the air. This is called *insensible* perspiration, and normally it amounts to about one pint per day. In hot weather the perspiration is poured out faster than it can evaporate, and it collects and runs down the skin as *sensible* perspiration. The total quantity of the perspiration varies inversely with the amount of urine secreted. In hot days when muscular activity produces increased heat, the skin is flushed with blood and the perspiration is greater, whereas the renal secretion is scanty. The circulation and amount of blood in the skin is controlled by the vaso-motor nerves through the vaso-motor centre under the influence of nervous excitation, and instances of these have already been given in the blushing of the face and its pallor under emotions, whereas the secretion of perspiration, on the other hand, is controlled by a special nervous apparatus of its own; the secretion being stimulated by changes in the quality and temperature of the blood, or by sensations of heat and cold, which are carried to the central nervous system through a set of afferent fibres, returning as impulses to secretion through another set of efferent fibres. It is thus seen how important a structure the skin is, and how necessary it is to understand the treatment of nervous, mental, and other diseases by massage, electricity, and hydrotherapy applied through the skin.

### Private Nursing in Russia.

BY AN ENGLISH NURSE.

It was in Hanover that I first saw my little Russian patient. She, with her mother and sisters, had been enjoying a few weeks' change at a German watering-place, and had come to Hanover because it was on their line of route back to Russia, and was a convenient halting-place for what the mother much desired to secure, an English nurse to return with her to Russia. The eyesight of the child—she was about ten years of age—had long caused the parents much anxiety, and at last it had been decided that before very long she must undergo an operation. About six weeks had passed since my last engagement terminated, and I had much enjoyed a restful stay with a very dear old friend. Very soon after I had agreed to accept the post offered we started for Riga, breaking our long journey for a day or two only in Berlin. Our party was sufficiently numerous to requisition an entire compartment, for which we were devoutly thankful, as thereby we were enabled to arrange ourselves just as we liked, and thus ensure some hours' sleep.

### Crossing the Frontier.

It was in the twilight of the second day that we crossed the frontier, went through the usual trouble and chance annoyances of the Custom House, and then plunged away through what seemed interminable forests of pine. At last the train pulled up alongside the Riga platform, and a short drive in drosches brought us home. The term Riga, at that

PRIVATE NURSING IN RUSSIA—*Continued.*

time, included the town proper of that name. and three suburbs—Petersbourger, Moscauer, and Mitauer Vorstädte. Three of these divisions were on the right side of the Dwina, the fourth, and last-mentioned, on the left of that river, being reached by means of a long iron bridge. Between the town proper and the other two suburbs stretched the promenades or boulevards, with a canal meandering through the centre providing exceedingly pleasant strolling and boating facilities for half the year, and, when the cold set in, means of enjoyment to skaters.

### Kindness of the Family Physician.

During the winter months we resided in a commodious and well-warmed flat, pleasantly situated and overlooking the boulevards, but in summer we migrated to the country house on the other side of the Dwina. Fortunately for me, the pending operation was not to take place till some few weeks after our return, for, during our journey north, I caught a severe chill and was myself for a time on the sick list. This gave me, however, an opportunity of personally testing the kindness and skill of the family physician. Under his careful treatment, into the details of which I need not enter, I made a speedy and complete recovery. I found that doctors were not paid the same in Russia as they are in England. Instead of the doctor's bill coming in at intervals, a certain fixed amount was regularly sent to him, in consideration of which he was expected to keep the family in good health, or, if any member became ill, to restore the same as quickly as possible. The days sped away quickly and happily. My duties were by no means onerous; I had plenty of leisure for necessary exercise and for recreation, and very frequently I assisted Madame S. in housekeeping matters—*e.g.* paying bills, ordering in stores, etc., as she was not very strong, and was glad to be relieved of the burden. She was, as Russians generally are, good at languages, and, being eager to know English, was glad to learn of me during the evenings. Her progress was very rapid—not attained by any cut-and-dried method, but by attentive listening, while I first read aloud a passage in English, then gave her the meaning in German or French, then re-read the passage in English, and so on. Then she would try, and before I left she both spoke and read English well.

### The Operation.

At last the time came for the operation, and my little patient and I repaired to the home of the specialist. Into the details of the preparation of the room I need not enter: all readers of The Hospital are but too well acquainted with these *minutiæ*. The child's mother very much wished to be present, but the doctors would not consent. To my surprise she persisted in waiting in the corridor close to the closed door. During the early stage of the proceedings I held the child's hand, as my touch seemed to give her courage. She had "gone off," and the skilled surgeon had just commenced, when a moan outside attracted the attention of the family doctor. He quietly suggested that I should go to the mother, which I did. I led her away and calmed her, and then returned in time to take charge of the child before she had sufficiently come round to notice my absence.

For three weeks we lived in the doctor's house, two weeks out of which time my little patient was in a perfectly darkened room, divided from mine by a very thick dark curtain. With the dressing of the eye I had nothing to do: every night and morning what was requisite was done by the doctor. This extra care resulted from a previous sad experience. The child's eyes had some time before been unsuccessfully operated upon, and this time no means were spared to ensure success. On me rested the responsibility of seeing that no bandage was removed, nor ray of light admitted until the fiat went forth from headquarters. Two hours daily Madame S. sat with my patient, while I went out for air and exercise. Our meals were good, well prepared, and well served. As far as I possibly could, I relieved the monotony for the child by telling stories, generally fairy tales, of which she was very fond and of which I had a good repertoire. It was fortunate for us both that I knew German and French. At that time Russian was not taught in the public schools of the Baltic Provinces, so that my little patient and I got on splendidly in the languages we both knew. The medical men were also conversant with these tongues, as in fact were all educated people in those districts, and so my ignorance of Russian never hampered me in the least. Only with quite the country market folk did I find any difficulty, and they spoke Lettisch, a dialect in some sounds provokingly like English, at least so Madame S. found it, and frequently, to her great annoyance, she would discover that she was sliding into my native tongue when talking with the market women. We grew very fond of one another during those days of isolation—a state of utter dependence often causes love to grow—and she was a dear child. Still, we were glad to get back into sunshine and movement again, and the absolute success of the operation made the return home a very glad one.

### The Winter Season.

Shortly after October opened, and with that month the Russian winter often begins—it did that year, and proved a specially hard season. From October till March skating was to the fore; wheels resigned their work to sleigh-runners, and the music of the bells was frequently the only perceptible noise in the white and glittering boulevards. The Dwina presented an especially pretty sight. Before the ice was quite "set" trees had been fixed avenue-wise from bank to bank, and these became encrusted with snow and hung with icicles. The iron bridge was temporarily neglected, and all traffic was carried on upon the frozen waters of the river. During the time I was in Russia I met with great and constant kindness and consideration, my "free" Sunday evenings being specially happy. One is apt, when far away from England, to get homesick, and this is usually worse on Sundays. I suppose the chaplain of the Anglican Church was aware of this fact, for he and his wife kept open house every Sunday night for the few English professional and unattached folk of both sexes at that time in Riga. In March the winter ended and the débâcle, or breaking-up of the ice, occurred—a sight well worth witnessing—the noise of wheels once more resounding in the thoroughfares.

### Another Patient.

Meanwhile my first patient had recovered strength and spirits, but her younger sister had passed into my charge, a severe and stubborn skin disorder necessitating great care and attention. So my stay in Russia was prolonged. Towards the end of May we left the flat and went into the country, where we daily enjoyed our dip in the Dwina, as the river flowed past our grounds, and bathing-houses had been erected on its banks. While in the country house I made my first acquaintance with mosquitoes. The little girl had well-nigh recovered when these insects viciously attacked her. They also thought it worth while to taste a foreigner's blood. Finding the remedy given for my patient very efficacious, I decided to try it to relieve my irritation instead of troubling the doctor for a personal prescription. Alas! blood-poisoning was the result, localised however by the care and skill of the doctor, who, though kindness and gentleness personified, reprimanded me as much as his fatherly heart would allow. For a fortnight I had to maintain a recumbent position, with bandages kept constantly moist, and my diet was strictly limited—limited, however, in a way that admirably suited my taste—"fruit, sauermilch, and cake; no tea, no meat, no coffee." The enjoyment of the first three more than made up for any deprivation in the exclusion of the latter. The food in Russia generally suited me well. I took exception to four things only—raw herring, raw ham, black bread, and caviar.

RUSSIAN SUPERSTITION.

At the end of the summer we returned to town, and I began to think of departure. In addition to the ordinary preparations for a journey, I had to take two unusual steps—to obtain a passport and to have my fortune told! The former, of course, I expected; the latter took me by surprise, but madame was so insistent on the evil that might accrue to the household if I declined, that I yielded. I took no share in the arrangements, but was simply a passive factor in the case. Cards, palms of the hands, and dregs of the teacup were all brought into use. Of what was said I remember two things only—one, that a very long journey lay before me. Naturally: was I not about to embark for Lubeck *en route* for Frankfort-on-Maine? But the soothsayer said "No" to that; it was to be a much longer journey later on, and "later on" I certainly did go to the far-away South Pacific. The second prognostication which I remember referred to something that was to transpire "very late in life," and possibly "very late in life" has not yet come to me. A recent statement in the daily papers that the Riga Fleet had been ordered to the Far East brought vividly back to mind my experiences in the Baltic, and enlisted my sympathy for those who should have to navigate those waters in wintry weather. It was early autumn when I embarked, but the weather was such that the genial captain assured me that, if I accomplished the voyage without a qualm, I might for ever consider myself proof against mal-de-mer. I passed through the ordeal unscathed, and have lived to prove the truth of the captain's prognostications.

### Ambidexterity in the Infant School.

A LECTURE, under the auspices of the Ambidextral Culture Society, was delivered by Miss Margaret McMillan at the rooms of the Medical Society, before rather a sparse audience, on Wednesday, March 2. The subject of the lecture was "Ambidexterity in the Infant School." Photographs of children engaged in drawing, some with the right hand, others with the left, and one or two with both at once, were handed round among the audience, as were also diagrams and drawings done by the tiny scholars, parts with the right hand and parts with the left. Miss McMillan showed that the "lopsidedness" of children was not altogether the result of training, as they came into the world having the right lung considerably heavier than the left. Experiments proved that there was a connection between the right hand and the left foot, and that right-handed people stepped more strongly on the left foot and rested upon it for choice, while the reverse was the case with left-handed people. The lecturer spoke very strongly upon the evils of the old system under which children learnt to write sitting at desks and forming small figures. The child should begin by making large letters with one hand or both, and it was most important that the left arm should not be neglected. Good nutrition and exercise of the weaker parts were necessary to ensure the perfect health of the whole system.

### Everybody's Opinion.

PORTRAITS AS PRESENTATIONS.

"C. H." writes: In the course of every year numerous testimonials are presented and gifts made by members of the nursing and other staffs of hospitals to their matrons and officers, and to popular sisters and numbers of others. Many of the recipients have done most excellent work in the institutions, or for the communities where they reside, and I would venture to propose that it would be a change for the better to present a well-painted miniature portrait to the matron or other recipient, instead of giving a present which can never have so permanent and personal an interest. The plan I suggest would have the further value that a replica of the portrait might be made and retained permanently in the board-room or the nurses' recreation-room, where in course of years a portrait-gallery of never-failing interest would be formed. Could you further this scheme, if you approve it, by undertaking to recommend a miniature painter, a woman of genius by preference, who would be prepared to co-operate, on reasonable terms, and so make this scheme practically possible?

*.* Highly approving the suggestion of "C. H.," we have made inquiries, and shall be glad to recommend a miniature painter, who is a lady in the first rank, and whose work promises to put her at the head of her profession. We think, if the scheme proves popular, that we may be able to arrange that each portrait shall cost about ten guineas and each replica five guineas. In inspecting the hospitals we have been struck by the great interest excited in a few institutions where the practice of preserving the portraits of the men and women who have promoted its success are hung in the board-room. "C. H.'s" idea would prevent the waste of much money, and promote the *esprit* attaching to Alma Mater. We shall be pleased to hear the views of our readers on this suggestion.—ED. THE HOSPITAL.

MAKING IT HARD FOR JUNIOR NURSES.

"A LATE SUFFERER" writes: I rejoice exceedingly to see the allusion which you make in your "Notes on News" in the last issue as to the treatment so often meted out to junior nurses by those who are their seniors in the wards, and are supposed to instruct them in their duties. Before entering on my present position, I was for a few months at one of the big London hospitals, and was a victim nearly the whole time of the "bullying" spirit of my superiors in knowledge. On one occasion, very early in my career, I was bidden to sterilise some instruments. Never having done such a thing before, I asked the sister how I ought to proceed. The directions were simple and emphatic—"Find out!"—and my supposed instructor turned on her heel and walked away. And it was not only myself to whom such treatment was given. All my fellow "pros." complained of the same want of consideration, one poor girl made herself quite ill one day when she was asked to get out the instruments for a special operation at which she had never been present before, and upon asking for further details was told curtly, "It was quite time she knew." No help was given her, two important items were forgotten, and the girl was, in consequence, severely reprimanded. In some hospitals it seems to me that sisters and nurses quite forget that there was ever a time in which they, too, were beginners, and had to be shown and instructed.

SIR WILLIAM BENNETT ON PRIVATE NURSING.

"A MATRON" writes: In reply to "A Dublin Nurse," I may say that I do not think she can have read my letter in your issue of the 20th very carefully, or she would have seen—if she had desired to do so—that my reference to institutions with untrained matrons was not in any way disparaging to untrained matrons as women, but only with regard to their ability to judge of a nurse's work from a professional standpoint. And that in those institutions a nurse might sometimes, under exceptional circumstances, be in some doubt about what is really right for her to do. If "A Dublin Nurse" finds my obvious meaning difficult to understand, I will take, for example, "two gentlemen," who may be equal with regard to educational advantages, social standing, and natural ability. But the fact of the one being a fully-qualified medical practitioner, and the other not, should make all the difference in their separate ability to thoroughly understand and give advice in medical matters. Even so a fully-trained matron should be in a better position to judge for her nurses in any case of doubtful propriety than an untrained one, because she has learnt by a long and practical experience to thoroughly understand "a patient's need" as well as a woman's natural delicacy of feeling, and also the true relation of a nurse to her patients. In reply to "A Frequent Contributor" I am sorry to say that I think her zeal has outrun her discretion in giving such prominence to her opinions, and laying down the law with regard to a branch of nursing which she knows absolutely nothing about except from hearsay. I also think that I am quite safe in saying that the great majority of our best and most experienced private nurses do not share her views. Many of them have for several years frequently nursed male patients in their greatest need and most utter helplessness without having lost one jot or tittle of their noble womanhood or the true gentlemanly respect of their patients. If a nurse does not degrade her profession her profession will never degrade her womanhood.

[Owing to the interest excited by Sir William Bennett's address it has been reprinted in pamphlet form under the title of "Modern Nursing in Private Practice," by the Scientific Press, Limited, 28 & 29 Southampton Street, W.C., price sixpence, post free sevenpence.—Ed. Hospital.]

### AN URGENT NEED.

Miss Mary E. Poole, hon. secretary of the Ladies' Auxiliary of the Dublin University Mission to Chhota Nagpur, writes from 15 Lower Fitzwilliam Street, Dublin: Will you allow me, through the medium of The Hospital, to lay before your Irish readers a very urgent need and a wide sphere of usefulness at present existing in the district assigned to the Dublin University Mission to Chhota Nagpur, Bengal, working under the Society for the Propagation of the Gospel? One of the distinctive features of this mission has been the medical work carried on from its commencement. There are at present three hospitals under the care of medical missionaries and the nurses of the staff. But two of the latter have resigned within the past few months, and a reinforcement is essential. It is hoped to open a hospital for women—a sore need—if the committee can secure the services of a lady doctor as well as a fully-trained nurse. We know that there are many Irish ladies at present studying in England for the medical and nursing professions, some of whom are doing so with the intention of dedicating their talents to missionary medical work. We desire most earnestly to ask them to consider the claims of this mission so closely linked with their own country, and, if free to do so, whether they will volunteer for work in this special part of the mission field. All information will be gladly given to any lady desiring further particulars.

### "ABIDE WITH ME."

Lady Maxwell Lyte, hon. secretary of the concert for the rebuilding fund of Brixham Church, who thanks us for an allusion to the appeal in our issue of February, writes: Two hundred pounds have been received in response to the appeal published early in February on behalf of the rebuilding of Lower Brixham Church, Devon, in memory of the author of "Abide with Me." But £2,000 is the sum needed for its completion, and the Vicar, the Rev. Stewart Sim, will be glad to acknowledge any sums sent to him for this purpose by lovers of the hymn. A matinée concert, under the patronage of Princess Christian, will be held on May 10th at Grosvenor House, and will be supported by many influential West Country ladies. Madame Clara Butt will sing "Abide with Me" at the concert, and twelve other famous artists have generously given their services. Tickets may be obtained from the hon. sec. of the concert, Lady Maxwell Lyte, 3 Portman Square, W.

### ALLEGED UNJUST TREATMENT.

"D. W." writes: After reading the case of alleged unjust treatment received by a charge nurse at an asylum a few weeks ago I wish to say that I think it most unfair that after she had been in charge of a most responsible ward for so long a time she should have received so little consideration both from matron and doctor. The very period of seven years' service ought to have merited a testimonial which would have given her a chance of securing another post. There is really no cause for wonder why many women take up the nursing and then abandon it. If resignations and applications were investigated by the committee there would be less cause for complaint.

### Appointments.

Bolingbroke Hospital, Wandsworth Common.—Miss P. G. Moores has been appointed staff nurse. She was trained at the Bethnal Green Infirmary, where she was afterwards staff nurse.

Carshalton Cottage Hospital.—Miss Letitia Bown has been appointed matron. She was trained at the National Orthopædic Hospital, London, and St. Mary's Hospital, Manchester. She has since been theatre and ward sister at St. Mary's Hospital, Manchester, sister at the General Infirmary, Chichester, and sister at the General and Eye Hospital, Swansea. She has also done private and district nursing, and holds the St. Mary's, Manchester, midwifery certificate.

Hereford General Infirmary.—Miss Margaret Borrie has been appointed night sister. She was trained at Gravesend Hospital.

Liverpool Queen Victoria District Nursing Association.—Miss Colvin has been appointed superintendent. She was trained at Brownlow Hill Infirmary, Liverpool, and has since been superintendent of the District Nurses' Home at Tunbridge Wells.

Moorcote Sanatorium, Eversley, Hants.—Miss Helen Cerise James has been appointed charge nurse. She was trained at Stapleton Infirmary, Fishponds, Bristol.

West Suffolk General Hospital, Bury St. Edmunds. Miss Ada Hambrook has been appointed sister of the male surgical and accident ward and out-patients' department. She was trained at Queen Charlotte's Lying-in Hospital, Marylebone, and the Royal Infirmary, Hull, where she has since been sister-in-charge of the children's ward.

### TRAVEL NOTES AND QUERIES.

By Our Travel Correspondent.

Normandy for French Conversation (Nancy).—Certainly if you go to a convent you must conform to the rules, but these are few and simple in establishments where boarders are taken. One invariably is, that you should not speak antagonistically of the religion of the nuns, and good taste would dictate that from your own hearts; and another usually is, that you should be in the house by 9 p.m. This last is usually relaxed if you mention beforehand that you want to make some special expedition. There is a convent at St. Servan, Brittany, address Madame la Superieure, Convent du Sacré Cœur; another at Bayeux, Convent des Bénédictines; another, which I know well, is Convent des Sœurs de la Croix, Tréquier, Brittany; another, Convent des Sœurs de la Charité de St. Louis, St. Geldas de Rhuys, Sarzeau, Morbihan. At all these places your pension terms would be about 28s. to 30s. a week. The nuns do not speak English, and you are not likely to meet your countrywomen unless it might be at Bayeux or St. Servan. Tréquier is quite inland, and reached from Guingamp.

### Echoes from the Outside World.

### The King's Movements.

OWING to slight indisposition, the King was prevented from keeping his engagement at St. Paul's Cathedral on Sunday morning, but, as no exposure to the outside air was involved, his Majesty was able to preside at the dinner party at Buckingham Palace on Saturday evening. The guests included a number of foreign ambassadors. On Monday the King drove out, and again on Tuesday. The indisposition may now be regarded as having passed.

### The Queen at St. Paul's Cathedral.

ALTHOUGH the King was unable to leave the house on Sunday, the Queen, in dismal weather attended St. Paul's Cathedral in order to be present at the Centenary Service of the Bible Society. There was no ceremony observed, but the Queen's landau was drawn by four horses, and there were postilions and riders. The only escort was a police patrol, who rode 300 or 400 yards ahead of the Royal equipage. The second carriage contained members of her Majesty's suite. A few minutes later the Prince and Princess of Wales followed in a closed carriage. The Royal party arrived at the west door of the Cathedral shortly before noon, and were met by the Bishop of London, the Dean and the Canons residentiary, who conducted her Majesty and Princess Victoria with the Prince and Princess of Wales to the places reserved for them under the dome. At the conclusion of the Anthem the sermon was preached by the Archbishop of Canterbury upon the text "And God said let there be light: and there was light, and God saw the light that it was good." A collection was afterwards made in aid of the Society.

### The Princess of Wales at a Rowton House.

AT the annual meeting of the Rowton Houses, Limited, last week, Sir Richard Farrant, the Chairman, mentioned that during the previous week the Princess of Wales, accompanied by the Bishop of London and the Rector of Whitechapel, paid a surprise visit one evening to the Rowton House in Whitechapel. They found the house in all respects orderly, and every man well behaved. The visitors stayed some time, and Sir Richard said that he believed the Princess of Wales was very much interested in all that she saw.

### A Royal Engagement.

THE KING declared on Monday his consent to a contract of matrimony between her Royal Highness Alexandra Louise Marie Olga Elizabeth Thérèse Vera, born Princess of Great Britain and Ireland, Duchess of Brunswick and Luneburg, daughter of his Royal Highness the Duke of Cumberland, and his Royal Highness Prince Friedrich Franz IV., Grand Duke of Mecklenburg-Schwerin.

### The New Ambassador at St. Petersburg.

SIR CHARLES SCOTT having retired from the diplomatic service, the post of his Majesty's ambassador at St. Petersburg—which he held since 1898—has been bestowed upon the Hon. Charles Hardinge, Assistant Under-Secretary of State for Foreign Affairs. Mr. Hardinge was born in 1858 and entered the diplomatic service in 1881. His first appointment was at Constantinople, and he has since served at Berlin, Washington, Sofia, Bukarest, Paris, Teheran, and St. Petersburg. He speaks Turkish, Persian, and Russian, among other languages. As his last appointment, before he became Assistant Under-Secretary of State in 1903, was that of Secretary of Embassy in St. Petersburg, he is well known in the capital to which he returns as chief. Mr. Hardinge married in 1890 the Hon. Winifred Selina Strutt, Woman of the Bedchamber to the Queen, and sister of the present Lord Abington.

### The War in the Far East.

THE Army orders issued on Saturday night contained the following order on the war between Russia and Japan:—"His Majesty having been pleased to publish a proclamation of neutrality in the war between Russia and Japan, general officers commanding will take steps to secure that the directions therein laid down are brought to the knowledge of those serving in their respective commands, and are strictly complied with." M. Plancy, the French Minister at Seoul, has thanked the Japanese Minister, in the name of Russia, for the humane treatment accorded to the wounded Russians.

Admiral Alexieff reports under date of March 7th that five of the Japanese ships on Sunday afternoon opened fire on Forts Suvaroff and Liniovitch at Vladivostock, and on the town and roadstead along the valley of the river Obyassneniye. The firing lasted for nearly an hour, when the Japanese vessels steamed to the southward. There were no losses in the batteries and fortifications, but in the town a seaman was wounded and a woman killed.

### Art Exhibition.

ON Saturday the Duchess of Albany opened the Royal Amateur Art Society's annual exhibition held this year at Moncoroo House, Ennismore Gardens. In addition to amateur work in painting, enamels, bookbinding, embroidery and other "arts and crafts," there was a loan collection of very choice works of a past age, which was confined on this occasion to the miniatures of two men, George Engleheart and John Smart, both very fully represented by some beautiful gems. In the class of amateur enamels Mrs. Dick obtained the prize, and Mrs. Bethune was very highly commended, but the finest things were sent by Mr. Gambier Howe, the prize-winner of the previous year, who was thus ineligible. In bookbinding Mrs. Moffat took the prize for a large volume bound in velvet with floral ornaments in gold and silver lace. Several excellent specimens of leather bindings, tooled and gilt were shown. Miss F. Rodd carried off the prize for watercolour, and Captain A. Fuller Maitland for oil-painting. Lady Victoria Herbert contributed some beautiful embroidery, and Princess Christian a painted screen. As in former years, the proceeds of the Exhibition will be divided amongst the Parochial Mission Women Fund, the East London Nursing Society, and the Deptford Fund.

### New Play at Daly's Theatre.

THE new musical play produced at Daly's Theatre is likely to be a considerable success. It possesses a plot of some interest, several cleverly-sketched characters, and is rich in songs which will delight all lovers of light melodies. The words for *The Cingalee* have been supplied by Mr. John Tanner, and the music by Mr. Lionel Monckton, who has called to his aid Messrs Adrian Ross, Percy Greenbank, and Paul Rubens. The scenery is particularly effective, and Mr. Hawes Craven has not done anything better than the "tea plantation" upon which the first act opens. The piece might have been accurately described as "In Search of a Pearl and a Girl," but this would probably have revealed too much of the leading theme. Mr. Hayden Coffin takes the part of the lover, Harry Vereker; Mr. Rutland Barrington is gorgeous as a powerful native nobleman, Boobhamba; Mr. Huntley Wright is amusing as a Baboo lawyer, Chambuddy Ram, whilst Miss Sybil Arundale makes a very pretty tea-girl, Nanoya, and Miss Gracie Leigh is the governess-adventurer who comes to help to fit Nanoya for her future position. The Queen and Princess Victoria were present on the opening night, and remained to the end of the performance.

### Notes and Queries.

**FOR REGULATIONS SEE PAGE 277.**

*One Year's Training.*

(208) Is there any possibility of a well-educated, intelligent, foreign girl of twenty-six being trained as a nurse in one year?—*M. N. F.*

She can take a course of training at the London Hospital, Whitechapel, E., or at the Middlesex Hospital, Mortimer Street, W., or at any other institution receiving paying probationers if she is prepared to pay about £1 1s. a week. Or through advertisement she may get a year's excellent teaching on reciprocal terms at one of the smaller hospitals which is not a recognised training school for nurses. One year's training, however, does not make a trained nurse.

*Private Imbecile Patients.*

(209) Is it necessary for an ex-attendant, who holds the Medico-Psychological certificate to obtain a license to keep as boarder an imbecile, or one mentally afflicted in a slight degree?—*W. W.*

No. But if you contemplate receiving mentally-afflicted boarders you would be well advised to write for the regulations of the Lunacy Board for England and Wales. Apply the Secretary, 66 Victoria Street, London, S.W.

*Maternity Training.*

(210) I am anxious to be trained for maternity work; can you tell me anything about a maternity training school at Acton? Is it a home for private cases, or are the pupils trained in a district?—*Taken-in.*

We cannot advise about particular homes.

*Visiting Nurses.*

(211) There was a most interesting account in your valuable paper some time ago of an association from which nurses could be obtained at a charge of 2s. 6d. an hour. Will you kindly tell me if there is such an association in existence?—*A. M. C.*

Probably you refer to the Marylebone Visiting Nursing Association; if so, Nurse Thomas, 10 Montague Place, W., will supply full particulars.

*Medical Attendance.*

(212) I am the caretaker; my wife nurse-matron of an Isolation Hospital. Unfortunately I caught a cold and developed bronchitis. I called upon the medical officer of the hospital for advice, and he ordered me to bed and attended me until I recovered. I had to find a substitute to do my work and pay him out of my salary; in the meantime I have received a bill for medical attendance and medicine from the medical officer. I have for many years been connected with similar institutions, where the staff, if ill, never paid for medical advice. Can you advise me?

We are afraid we cannot help you. The medical officer appears to be quite within his rights in asking for remuneration for his trouble. Your grievance should be directed to the authorities of the hospital, with whom rests the responsibility for attending to the welfare of their servants.

*Fever Hospitals.*

(213) Will you please give me the names of fever hospitals in London?—*A. L.*

Apply to the Clerk to the Metropolitan Asylums Board, Victoria Embankment, E.C.

*Massage.*

(214) Can you tell me if I can learn massage in Belfast, or if a certificate could be obtained here (Barnsley)? I shall be much obliged for any information on the subject.—*K. M. W.*

There are no public schools in either place, but the Incorporated Society of Trained Masseuses, 12 Buckingham Street, Strand, W.C., would probably advise you.

### Standard Nursing Manuals.

"The Nursing Profession: How and Where to Train." 2s. net; 2s. 4d. post free.

"Nursing: Its Theory and Practice." (Revised Edition). 3s. 6d. post free.

"Elementary Anatomy and Surgery for Nurses." By William McAdam Eccles, M.D.Lond., M.B. 2s. 6d. post free.

"Elementary Physiology for Nurses." By C. F. Marshall, M.D., B.Sc., F.R.C.S. 2s. post free.

"Ophthalmic Nursing." By Sydney Stephenson, M.B., F.R.C.S., 3s. 6d. net; 3s. 10d. post free.

"Nursing in Diseases of the Throat, Nose, and Ear." By P. Macleod Yearsley, F.R.C.S.Eng., M.R.C.S. 2s. 6d. post free.

"Fevers and Infectious Diseases." 1s. post free.

### For Reading to the Sick.

"ALL FULNESS DWELLS IN THEE."

I AM so needy, Lord, and yet I know
All fulness dwells in Thee;
And hour by hour that never-failing treasure
Supplies and fills, in overflowing measure,
My least, my greatest need; and so
Thy grace is enough for me!

It is so sweet to trust Thy Word alone;
I do not ask to see
The unveiling of Thy purpose, or the shining
Of future light on mysteries untwining:
Thy promise-roll is all my own,—
Thy Word is enough for me!

*F. R. Havergal.*

When therefore the smallest instinct or desire of thy heart calleth thee towards God, and a newness of life, give it time and leave to speak; and take care thou refuse not Him that speaketh. . . . Be retired, silent, passive, and humbly attentive to this new risen light within thee.

*Wm. Law.*

And the sick have many chances for its exercise, and need it emphatically. They need it for themselves, to restrain the impatience and restlessness of soul to which protracted suffering naturally gives rise. At a time of sickness and convalescence, their souls are a scene of great activity; many forces are at work, and they need the gentle restraint, the guiding hand of meekness to keep them in loving submission to God's holy will, to prevent all fretfulness, and to secure their peace. Moreover the sick have great influence for good over others; they have unusual facilities for "possessing the land" of other souls and winning them more fully to God.

But there is another land that meek souls possess: it is the land of the souls of others. Meekness is the mainspring of our influence for good over others, even as the want of it destroys all power of helping them or of winning their love. Meekness, watered by charity, is a passport to the confidence of others. At peace ourselves, we bring others to peace, and calm their restless spirit, and set them on the road to spiritual health and vigour.

"Submit thyself, O my soul, to God and be at peace; thereby thou shalt have the best fruits, and the peace of God which surpasseth all understanding shall keep thy mind and heart in Christ Jesus."—*Anon.*

Teach us, Master, how to give
All we have and are to Thee;
Grant us, Saviour, while we live,
Wholly, only, Thine to be.
Henceforth be our calling high,
Thee to serve and glorify;
Ours no longer, but Thine own,
Thine for ever, Thine alone.

*F. R. Havergal.*

